# Supplementary material for: Technology in farming: Unleashing farmers’ behavioral intention for the adoption of agriculture 5.0
Source: PLoS One. 2024 Aug 22;19(8):e0308883. doi: 10.1371/journal.pone.0308883 (PMC11340900; doi:10.1371/journal.pone.0308883)
Supplement: Attachment — Submitted filename: Review on TAM on Farmer Behahioral.docx [file pone.0308883.s001.docx]

**Review on PONE-D-23-25144R1**

**Title:** Technology in Farming: Unleashing Farmers’ Behavioral Intention for the Adoption of Agriculture 5.0

**Overall, the author and the crew effectively respond to all the criticisms raised by the reviewers. The provided justifications were both reasonable and acceptable.**


Specific feedback:

1.	Kindly provide an introductory statement for each of the subtopics. For instance, on page 3, in the methodology section, begin by providing a preamble and then proceed to explore the following topics.

2.	On page 9, the author continues to employ purposive sampling.

3.	What about employing G-Power for sample determination?
