# Supplementary material for: Technology in farming: Unleashing farmers’ behavioral intention for the adoption of agriculture 5.0
Source: PLoS One. 2024 Aug 22;19(8):e0308883. doi: 10.1371/journal.pone.0308883 (PMC11340900; doi:10.1371/journal.pone.0308883)
Supplement: Attachment — Submitted filename: Response to Reviewers R2.docx [file pone.0308883.s002.docx]

**Response to Reviewers**

Dear Editor, Greetings form Nepal!

Thank you for providing us with the minor corrections on our manuscript. We are pleased to have satisfied our reviewer with our responses to their queries and suggestions in the R1 phase.

After thorough discussions among the team and authors, and carefully reviewing the comments, we have prepared detailed responses to each query. Some comments were aimed at improving our understanding rather than requiring manuscript changes, and some suggestions were not feasible due to the completion of the process. Therefore, we have provided rebuttals where necessary. Please understand that it is challenging to redo every step of the process entirely after correcting R1 from the journal. We tried our best to cover every comment as much as possible.

We have included a tracked changes version and a final version of the manuscript to ensure transparency. We hope this helps the reviewer to re-check their feedback and confirm that all issues have been addressed.

Table 1: Response to reviewers – point by point

| **Additional Editor Comments** |  |
| --- | --- |
| Dear Author Thank you so much for your effort. The manuscript can be accepted after robustness check of your model. Thank you. | Dear Editor,  Thank you for your kind words. This is very good news for researchers like us. As per your suggestion, we have performed the robustness check on our model and reported the results in the manuscript. The details can be found in the Results section.  Thank you very much. |
| **Journal Requirements** |  |
| Please review your reference list to ensure that it is complete and correct. If you have cited papers that have been retracted, please include the rationale for doing so in the manuscript text, or remove these references and replace them with relevant current references. Any changes to the reference list should be mentioned in the rebuttal letter that accompanies your revised manuscript. If you need to cite a retracted article, indicate the article’s retracted status in the References list and also include a citation and full reference for the retraction notice. | Dear Editor,  We have reviewed our reference list to ensure it is complete and correct. We have replaced any cited papers that have been retracted with relevant current references. All suggested changes have been made, and the reference list is now accurate and up-to-date.  Thank you very much. |
| **Reviewers' comments:** |  |
| **Reviewer #1:** |  |
| Hi, Pleas double check the reference list that all of the references were cited correctly. For example I saw that one of the references was not cited completely that is needed to be corrected as below:  Salehi, S., Rezaei-Moghaddam, K., & Ajili, A. (2012). Extension of grid soil sampling technology: application of extended Technology Acceptance Model (TAM). Journal of Research in Agriculture. 1, 078-087. | Dear Rezaei-Moghaddam,  Thank you for pointing out the issue with the reference. We have now corrected the reference as suggested:  Salehi, S., Rezaei-Moghaddam, K., & Ajili, A. (2012). Extension of grid soil sampling technology: application of extended Technology Acceptance Model (TAM). Journal of Research in Agriculture, 1, 078-087.  We have double-checked the entire reference list to ensure all references are cited correctly. |
| **Reviewer #3:** |  |
| Thank you so much for submitting the revised version of the manuscript. Previous comments have been mostly addressed. However, although the data normality test has been performed, the data robustness check still lacks sufficiency. In the PLS-SEM approach, robustness check can be performed in three ways:  1. the non-linear effects 2. Unobserved heterogeneity 3. Endogeneity | Dear Riffat Ara Zannat Tama,  Thank you for your kind words and appreciation of our previous revisions. Your feedback has been invaluable in helping us improve our manuscript.  As per your suggestion, we have now performed the data robustness checks in the context of our dataset using the three recommended methods: non-linear effects, unobserved heterogeneity, and endogeneity. These analyses are thoroughly discussed and included in the Results section of the manuscript.  Thank you once again for this opportunity to learn and improve, as we have not previously utilized these robustness checks in our earlier papers. |
| **Reviewer #4:** |  |
| Thank you very much for giving me the opportunit to review the paper! I am sorry to say that ins current form the paper cannot be accepted for publication. In fact, I recommend the paper to be rejected. First of all, the paper does not provide a clear research gap or its contribution to the literature. Second, the framework is random and falsely used. In consequence, the implications given by the results are not valid. Hence, the addition of the paper to the literature is questionable. | Dear Reviewer,  Thank you for your valuable feedback on our paper. We have carefully considered each point raised and discussed them thoroughly among all authors. We revised the introduction and literature review sections to clearly articulate the specific research gap our study addresses, and revisited our theoretical framework to ensure it aligns with our research objectives. We also re-examined the results and their implications, performing robustness checks to validate our findings. We believe these revisions significantly strengthen the paper and address the issues you identified. We kindly request you to review the revised manuscript and provide any further feedback. Thank you. |
| Major points: The introduction is not clearly written and understandable. The authors jump back and forth between various themes. For instance, they start explaining on page 2 the concept of Ag 5.0 and then go back to the Green Evolution and then again Ag 5.0. Hence, there is no clear structure in the introduction and therefore the research gap is unclear. I was not able to detect based on the literatur provided what is the research gap and what is the contribution of this paper. This needs to be backed up by literature. Furthermore, for the authors it is not of interest, what the authors belief (p. 3) regarding farmers' beliefs. This needed to be backed up by literature. Hence, the complete introduction needs to be rewritten. | Dear Reviewer, thank you once again.  As per the suggestion, we author cross check the introduction section make necessary correction as it requires. |
| The framework is very unclear. Why does the authors try to combine elements from the UTAUT (Facilitating conditions, Social Influence) and the TAM (Perceived Usefulness)? The UTAUT is already as stated by its name (Unified Theory of Acceptance and Use of Technology) the essence of 8 distinct theories, including the TAM. Hence. the framework feels random, even though back up by another reference. It is very questionable why the authors have not chosen to follow the path of the UTAUT and extend it with technology anxiety. To highlight this puzzling choice by the authors: According to the UTAUT, it is expected that Social Influence has an an effect on the Intention to use Technology. Here, it influences Perceived Ease of Use and Perceived Usefulness. | Dear Reviewer, thank you once again.  As per the suggestion, we author cross check framework and make necessary correction as it requires. Some comments raised here is undertaken as knowledge for our next paper and issues as it is almost impossible to change for this model. We appreciate the reviewer’s comment and concerned on this part. |
| The part on Structural equation modelling misses most important parts regarding interal and external validity of the model. Furthermore information is missing on the used statistical software. | Dear Reviewer, thank you once again.  As per the suggestion, we author discuss regarding the internal and external validity and confirm it is prepare under the criterion needed. To strengthen external validity robustness test such as the non-linear effects, Unobserved heterogeneity and Endogeneity were test and add in manuscript. I hope this issue is added. Further, the use of statistical software is added as “The collected data was finalized and managed using Microsoft Excel, and final inferential data analysis was done using SPSS-AMOS software and SMART PLS 4.0” in method section. |
| Regarding the results, looking at the Cronbachs Alpha and Composite reliabiltiy. The values for Behavioral Intention exceed 0.95 which usually points semantically to close statements. Hence, these statements do not measure a common construct but just represent the same sentence all over again. For discriminant validity, it is always recommended to provide the CI95 value, which is missing. Effect sizes f2 are missing. Furthermore, the authors rely only statistical significance. Most effect sizes of the path coefficient can be considered to be very low. Hence, the practical relevance is missing and not discussed by the authors sufficiently. Lastly, as usual one should test for quadrat effects and endogeneity via Gaussian copulas in SEM. Both is missing. | Dear Reviewer, thank you once again.  As per the suggestion, authors discuss and decided to add requested modification. Now, test for quadrat effects and endogeneity via Gaussian copulas in SEM is presented. Some comments are pertinent but we decided to keep it as it is due to unable to change it, if we change it whole stricture will be collapse. Some very important suggestion is definitely a great learning to us which we can reflect with our future work. |
| Minor points  Term significant should only be used in connection with statistical significane and inference. Figure 4: Barriers are not fully written out (Lack of Adquate Infrastructure &..). | Dear Reviewer, thank you once again.  As per the suggestion, term significance is replaced with suitable words as per its necessity and suitability.  The missing part of “Lack of Adquate Infrastructure &..” in figure 4 is now well prepared. |
| **Specific feedback:** |  |
| Kindly provide an introductory statement for each of the subtopics. For instance, on page 3, in the methodology section, begin by providing a preamble and then proceed to explore the following topics. | Dear sir,  Thank you for your feedback. As per your suggestion, we have provided introductory statements for each of the subtopics, including the methodology section on page 3. We believe these additions help to enhance the clarity and coherence of the manuscript.  Thank you for your guidance. |
| On page 9, the author continues to employ purposive sampling. | Dear sir,  Thank you for the feedback. The rationale for selecting purposive sampling is adequately addressed on page 9 of the manuscript. |
| What about employing G-Power for sample determination? | Dear sir,  Thank you for your feedback. We acknowledge the value of using G-Power for sample size determination. However, due to current limitations, we were unable to employ it for this study. We have reviewed G-Power and plan to incorporate it in our future research.  Thank you once again for your valuable suggestions. |

Thank you for your understanding and support.

Best regards,

Niranjan Devkota, PhD
